# Genetic Detection of *Candida albicans* and Measurement of IL-17 Levels in Rheumatoid Arthritis Patients: A Study Using Conventional PCR and ELISA Techniques

**DOI:** 10.3390/ijms27156831

**Published:** 2026-07-30

**Authors:** Abdullah AbdulKareem Ahmed Al-Obaidi, Farah Thamer Samawi, Mehmet Gökhan Halıcı

**Affiliations:** 1 Biological Research Unit for Tropical Regions, Faculty of Science, University of Baghdad, Baghdad 10071, Iraq; abduallaabdalkraeem@gmail.com; 2 Department of Biology, Graduate School of Natural and Applied Sciences, Erciyes University, Kayseri 38039, Türkiye; 3 High Institute of Forensic Sciences, Al-Nahrain University, Baghdad 64074, Iraq; farahthamer1978@nahrainuniv.edu.iq; 4 Department of Biology, Faculty of Science, Erciyes University, Kayseri 38039, Türkiye

**Keywords:** rheumatoid arthritis, *Candida albicans*, IL-17A, Th17, conventional PCR, ELISA

## Abstract

Rheumatoid arthritis (RA) is a chronic autoimmune disease characterized by persistent inflammation and progressive joint destruction. Increasing evidence suggests that fungal dysbiosis may influence immune responses through the interleukin-17A (IL-17A) pathway. This study aimed to determine the prevalence of peripheral blood *Candida albicans* DNA in patients with RA, quantify serum IL-17A concentrations, and evaluate the association between fungal DNA positivity and IL-17A levels. A total of 100 participants, including 50 patients with RA and 50 healthy controls, were enrolled. *Candida albicans* DNA was detected by conventional polymerase chain reaction (PCR) targeting the **INT1** gene, while serum IL-17A concentrations were measured using enzyme-linked immunosorbent assay (ELISA). The association between fungal DNA positivity and serum IL-17A concentrations was assessed using point-biserial correlation analysis. *Candida albicans* DNA was detected significantly more frequently in RA patients than in healthy controls (38% vs. 10%; *p* < 0.001). Serum IL-17A concentrations were significantly higher in RA patients, with the highest levels observed in *C. albicans*-positive patients (46.2 ± 8.7 pg/mL), followed by *C. albicans*-negative RA patients (32.5 ± 6.4 pg/mL) and healthy controls (15.1 ± 5.2 pg/mL) (*p* < 0.001). Point-biserial correlation analysis demonstrated a significant positive association between *C. albicans* DNA positivity and serum IL-17A concentrations (*r_pb_* = 0.672, *p* < 0.001). Peripheral blood *Candida albicans* DNA positivity was associated with elevated serum IL-17A concentrations in patients with RA. These findings support an association between fungal DNA detection and enhanced IL-17A-mediated immune responses; however, the cross-sectional design precludes any inference of causality. Further longitudinal studies incorporating quantitative fungal detection are warranted.

## 1. Introduction

Rheumatoid arthritis (RA) is a chronic systemic autoimmune disease characterized by persistent synovial inflammation, progressive cartilage destruction, bone erosion, and irreversible joint disability. The pathogenesis of RA is multifactorial and involves a complex interplay among genetic susceptibility, environmental exposures, epigenetic modifications, and dysregulated innate and adaptive immune responses [1,2]. Despite considerable advances in understanding the immunopathogenesis of RA, the precise mechanisms underlying disease initiation and progression remain incompletely understood. Increasing evidence indicates that the human microbiome, including both bacterial and fungal communities, plays a crucial role in maintaining immune homeostasis and may influence the development and persistence of autoimmune diseases through interactions with mucosal and systemic immunity [3,4,5,6].

Among fungal microorganisms, *Candida albicans* is the most prevalent opportunistic commensal fungus colonizing the oral cavity, gastrointestinal tract, and other mucosal surfaces. Although it generally exists as a harmless commensal in healthy individuals, disruption of mucosal barrier integrity, immune dysregulation, or immunosuppressive therapy may facilitate fungal overgrowth or promote the translocation of fungal cells or fungal DNA into the circulation [7,8,9]. Recent clinical and experimental studies have demonstrated that fungal dysbiosis is associated with chronic inflammatory disorders, including RA, suggesting that altered host–fungus interactions may contribute to sustained immune activation rather than acting as an independent causal factor [4,5,6,10].

One of the principal immunological pathways linking *C. albicans* to inflammatory diseases involves activation of T helper 17 (Th17) cells. Recognition of fungal cell-wall components by dendritic cells stimulates the production of interleukin-23 (IL-23) and other cytokines that promote Th17 differentiation and the subsequent secretion of interleukin-17A (IL-17A) [11,12,13]. IL-17A plays an essential role in antifungal host defense by recruiting neutrophils and enhancing mucosal immunity. However, persistent activation of this pathway has also been implicated in the pathogenesis of RA, where excessive IL-17A production promotes chronic synovial inflammation, osteoclast differentiation, cartilage degradation, and bone erosion through interactions with other pro-inflammatory cytokines, including tumor necrosis factor-alpha (TNF-α) and interleukin-6 (IL-6) [14,15,16,17]. Recent clinical and experimental evidence further suggests that intestinal fungal dysbiosis may enhance Th17-mediated immune responses and aggravate inflammatory arthritis, although the underlying biological mechanisms remain incompletely understood [5,6].

Because *C. albicans* is a potent physiological inducer of Th17 differentiation, an association between fungal colonization, fungal DNA detection, and increased IL-17A production is biologically plausible [11,12,13]. Nevertheless, the detection of fungal DNA in peripheral blood (DNAemia) should not be interpreted as evidence of active fungal infection or systemic candidiasis. PCR-based detection may reflect transient microbial translocation across compromised mucosal barriers, circulating DNA derived from non-viable fungal cells, asymptomatic colonization, or, less commonly, laboratory contamination [18,19]. Consequently, fungal DNA positivity should be interpreted cautiously within its clinical context, particularly because low levels of fungal DNA may occasionally be detected in apparently healthy individuals.

Several hypotheses have been proposed to explain the interactions between microorganisms and autoimmune diseases. One proposed mechanism is molecular mimicry, whereby microbial antigens share structural similarities with host proteins, potentially contributing to the loss of immunological tolerance. Although this hypothesis has been investigated in several autoimmune disorders, direct evidence supporting molecular mimicry specifically between *Candida albicans* and rheumatoid arthritis remains limited. Therefore, this mechanism should currently be regarded as speculative and requires further experimental validation before any causal role can be inferred [10,20].

Recent studies have increasingly emphasized the importance of the fungal microbiome (mycobiome) in regulating immune responses and maintaining mucosal homeostasis. Alterations in fungal community composition, impaired epithelial barrier integrity, and immune dysregulation may collectively increase exposure of the host immune system to fungal antigens, thereby sustaining inflammatory responses [4,5,6]. Conversely, chronic inflammation, reduced salivary flow, impaired oral hygiene resulting from joint disability, prolonged antibiotic exposure, and immunosuppressive therapies commonly prescribed for RA—including disease-modifying antirheumatic drugs (DMARDs), corticosteroids, and biological agents—may themselves increase susceptibility to *Candida* colonization or fungal DNA translocation [8,9,21]. Therefore, the relationship between *C. albicans* and RA is likely to be bidirectional rather than causal, and both immunological and non-immunological factors should be considered when interpreting fungal DNA positivity in patients with RA.

Accordingly, the present study aimed to determine the prevalence of *Candida albicans* DNA in peripheral blood samples obtained from patients with rheumatoid arthritis using conventional polymerase chain reaction (PCR), quantify serum IL-17A concentrations using enzyme-linked immunosorbent assay (ELISA), and evaluate the association between fungal DNA detection and circulating IL-17A levels. Rather than establishing a causal relationship, this study seeks to improve our understanding of the association between fungal DNAemia and IL-17-mediated immune responses in patients with RA by integrating molecular and immunological findings.

## 2. Results

### 2.1. Study Population

A total of 100 participants were included in this study, comprising 50 patients with rheumatoid arthritis (RA) and 50 healthy controls (HC). The baseline demographic and clinical characteristics of the study population are summarized in Table 1.

The mean age was **48.3 ± 10.1 years** in the RA group and **46.7 ± 9.8 years** in the HC group, with no statistically significant difference between the groups (***p* = 0.421**). Female participants constituted the majority in both groups, accounting for **38 patients (76%)** in the RA group and **36 individuals (72%)** in the HC group. The sex distribution was comparable between the two groups (***p* = 0.643**). Among patients with RA, the mean disease duration was **7.2 ± 3.6 years**.

Overall, the RA and HC groups were well matched with respect to age and sex. However, detailed clinical and disease activity variables, including the Disease Activity Score in 28 joints (DAS28), C-reactive protein (CRP), erythrocyte sedimentation rate (ESR), rheumatoid factor (RF), anti-cyclic citrullinated peptide antibodies (ACPA), and medication history, were not consistently available for all participants and were therefore not included in the comparative statistical analyses. This limitation was taken into consideration when interpreting the associations between fungal DNA detection and serum IL-17A concentrations.

### 2.2. Prevalence of Candida albicans

The prevalence of peripheral blood *Candida albicans* DNA differed significantly between patients with rheumatoid arthritis (RA) and healthy controls (HC). In the RA group, 19 of 50 patients (38%) tested positive for *C. albicans* DNA, whereas only 5 of 50 healthy controls (10%) were positive.

**Figure 1:**
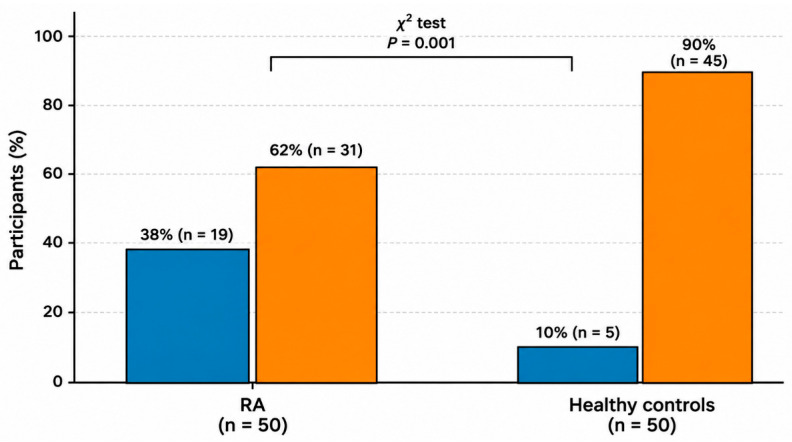
Prevalence of peripheral blood *Candida albicans* DNA positivity in patients with rheumatoid arthritis (RA) and healthy controls, determined by conventional PCR. Bars represent the percentage of participants who tested positive or negative for *C. albicans* DNA. Statistical significance was assessed using the chi-square (χ^2^) test (*p* = 0.001).

As shown in Table 2 and Figure 1, the frequency of *C. albicans* DNA positivity was significantly higher in patients with RA than in healthy controls (*p* = 0.001, chi-square test). Conversely, *C. albicans* DNA was not detected in 31 RA patients (62%) and 45 healthy controls (90%).

These findings indicate that peripheral blood *C. albicans* DNA was detected more frequently in patients with RA than in healthy individuals. However, because conventional PCR is a qualitative method that detects the presence or absence of fungal DNA, these findings should not be interpreted as evidence of active fungal infection or as a measure of fungal burden.

### 2.3. Serum IL-17A Levels

**Figure 2:**
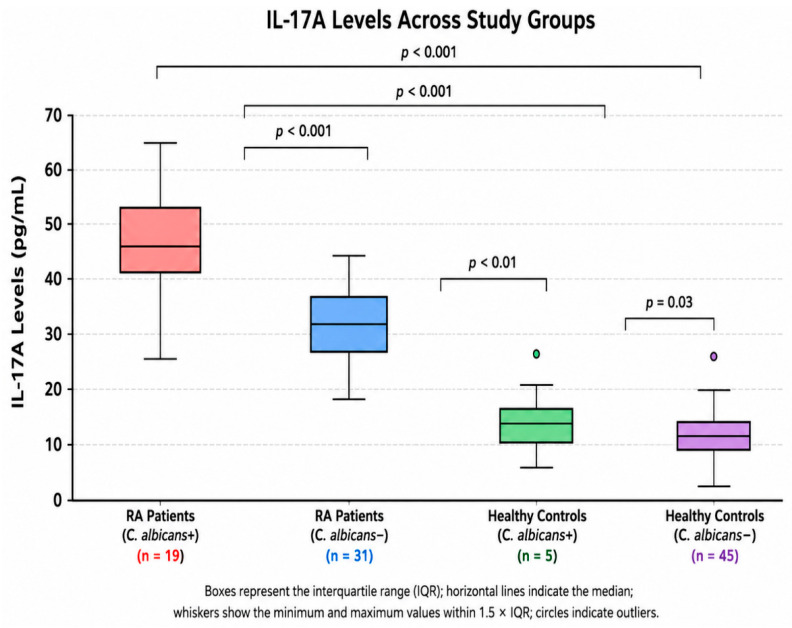
Distribution of serum IL-17A concentrations in RA patients positive for *Candida albicans* DNA (n = 19), RA patients negative for *Candida albicans* DNA (n = 31), healthy controls positive for *Candida albicans* DNA (n = 5), and healthy controls negative for *Candida albicans* DNA (n = 45). Boxes indicate the interquartile range, horizontal lines represent the median, whiskers indicate the data range within 1.5 × IQR, and circles represent outliers.

Serum IL-17A concentrations were compared among patients with rheumatoid arthritis (RA) who tested positive or negative for *Candida albicans* DNA and healthy controls (Table 3, Figure 2). The highest mean IL-17A concentration was observed in *C. albicans*-positive RA patients (46.2 ± 8.7 pg/mL, n = 19), followed by *C. albicans*-negative RA patients (32.5 ± 6.4 pg/mL, n = 31). Healthy controls (n = 50) exhibited the lowest mean serum IL-17A concentration (15.1 ± 5.2 pg/mL).

Compared with healthy controls, serum IL-17A concentrations were significantly higher in both *C. albicans*-positive RA patients (*p* < 0.001) and *C. albicans*-negative RA patients (*p* < 0.001). Furthermore, *C. albicans*-positive RA patients exhibited significantly higher IL-17A concentrations than *C. albicans*-negative RA patients (*p* < 0.001).

The distribution of serum IL-17A concentrations is illustrated in Figure 2. The median and interquartile range were highest in the *C. albicans*-positive RA group, intermediate in the *C. albicans*-negative RA group, and lowest in the healthy control group. Although some overlap between individual measurements was observed, the overall distribution demonstrated progressively higher serum IL-17A concentrations in both RA groups than in the healthy controls.

### 2.4. Association Between Candida albicans DNA Positivity and Serum IL-17A Concentrations

Because *Candida albicans* DNA positivity is a dichotomous variable, the association between fungal DNA status and serum IL-17A concentrations was assessed using point-biserial correlation analysis. Among patients with RA, *C. albicans* DNA positivity was significantly associated with elevated serum IL-17A concentrations (*r_pb_* = 0.672, *p* < 0.001).

**Figure 3:**
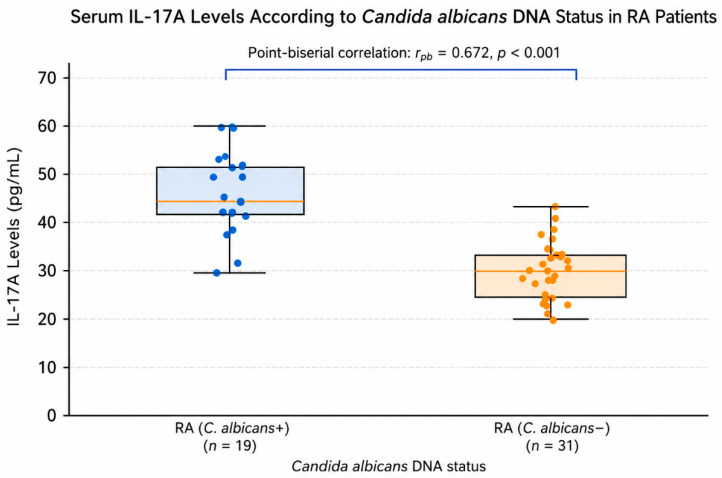
Association between *Candida albicans* DNA status and serum IL-17A concentrations among patients with rheumatoid arthritis. Individual data are presented according to PCR status. The association was evaluated using point-biserial correlation analysis (*r_pb_* = 0.672, *p* < 0.001).

As shown in Figure 3, RA patients who tested positive for *C. albicans* DNA exhibited higher serum IL-17A concentrations than those who tested negative. These findings demonstrate a statistically significant association between fungal DNA positivity and circulating IL-17A concentrations in patients with RA. However, given the cross-sectional nature of the study, this association should not be interpreted as evidence of a causal relationship.

### 2.5. Representative PCR Amplification of Candida albicans

**Figure 4:**
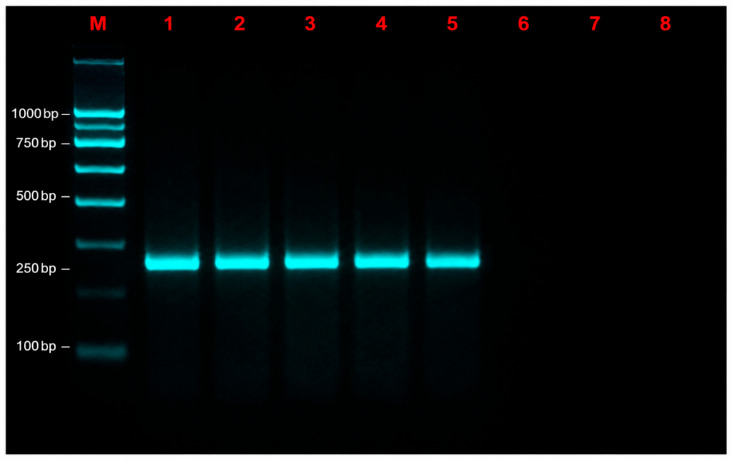
Representative agarose gel electrophoresis image showing amplification of the *Candida albicans* INT1 gene (344 bp). Lane M, 100 bp DNA ladder; Lanes 1–5, representative PCR-positive RA samples; Lanes 6–8, representative PCR-negative healthy control samples. The gel illustrates representative amplification patterns and is not intended to reflect the overall prevalence of PCR positivity observed in the study population.

Figure 4 shows a representative agarose gel electrophoresis image of PCR amplification targeting the *Candida albicans* INT1 gene. Distinct bands at approximately 344 bp were observed in lanes 1–5, representing *C. albicans* DNA-positive RA patients, confirming successful amplification of the target fragment. In contrast, no amplification products were detected in lanes 6–8, representing *C. albicans* DNA-negative healthy controls.

This representative gel image illustrates the specificity of the PCR assay used in the present study and demonstrates the expected amplification pattern for positive and negative samples. As this image is presented for illustrative purposes only, it should not be interpreted as reflecting the overall prevalence of *C. albicans* DNA positivity in the study population.

## 3. Discussion

This study investigated the association between peripheral blood *Candida albicans* DNA detection and serum IL-17A concentrations in patients with rheumatoid arthritis (RA). The findings showed that *C. albicans* DNA was detected significantly more frequently in patients with RA than in healthy controls and that serum IL-17A concentrations were significantly higher in the RA group, particularly among individuals with detectable fungal DNA. Furthermore, point-biserial correlation analysis revealed a significant positive association between *C. albicans* DNA positivity and elevated serum IL-17A concentrations. Although these findings support an association between fungal DNA detection and enhanced inflammatory responses in RA, the cross-sectional nature of this study precludes any conclusions regarding causality or the temporal relationship between these events [1,2,3,4].

Accumulating evidence indicates that the human microbiome, including the fungal microbiome (mycobiome), plays a critical role in immune regulation and the pathogenesis of autoimmune diseases. Although bacterial dysbiosis has been extensively investigated in RA, increasing attention has recently been directed toward fungal communities because of their capacity to modulate both innate and adaptive immune responses [5,6,7,8]. Experimental studies have shown that *C. albicans* activates dendritic cells through pattern-recognition receptors, including Dectin-1 and Toll-like receptors, thereby stimulating IL-23 production and promoting the differentiation of T helper 17 (Th17) cells, which subsequently produce IL-17A and other pro-inflammatory cytokines [9,10,11,12]. Because IL-17A contributes to synovial inflammation, osteoclast activation, cartilage degradation, and bone erosion, this immunological pathway provides a biologically plausible mechanism underlying the association between fungal DNA positivity and elevated serum IL-17A concentrations observed in this study. Nevertheless, these mechanistic insights are derived primarily from experimental and translational studies and should not be interpreted as direct evidence that fungal DNA detected in peripheral blood is responsible for increased IL-17A production in patients with RA [9,10,11,12,13].

Our findings are also consistent with those reported by Bishu et al. [22], who demonstrated that patients with rheumatoid arthritis exhibit impaired *Candida albicans*-specific Th17 responses despite preserved systemic immune activation. Their study suggested that dysregulated antifungal immunity in RA may alter host responses to *C. albicans*, potentially contributing to persistent fungal colonization and immune imbalance. Although the present study did not evaluate antigen-specific Th17 cell function, the observed association between peripheral blood *C. albicans* DNA positivity and elevated serum IL-17A concentrations supports the concept that altered host–fungus interactions may be involved in the immunopathology of rheumatoid arthritis. Nevertheless, because the two studies evaluated different immunological endpoints, direct comparisons should be interpreted with caution [22].

An important consideration when interpreting these findings is that the detection of *Candida albicans* DNA in peripheral blood should not be regarded as direct evidence of active fungal infection or invasive candidiasis. PCR-based assays detect fungal nucleic acids but cannot distinguish between viable and non-viable organisms or determine whether fungal DNA originates from active infection, transient microbial translocation, asymptomatic mucosal colonization, or circulating DNA fragments. Therefore, the observed DNAemia most likely reflects increased exposure of the host immune system to fungal components rather than disseminated candidiasis [14,15,16,17]. This interpretation is further supported by the detection of *C. albicans* DNA in a small proportion of healthy controls (10%), indicating that low-level fungal DNAemia is not exclusive to RA and may occasionally occur in apparently healthy individuals.

Several mechanisms may account for the higher prevalence of fungal DNA positivity observed in patients with RA. Chronic systemic inflammation has been associated with impaired epithelial barrier integrity in the oral cavity and gastrointestinal tract, thereby facilitating microbial translocation into the circulation [18,19]. In addition, long-term immunomodulatory therapy, including disease-modifying antirheumatic drugs (DMARDs), corticosteroids, and biological agents, may alter host defense mechanisms and increase susceptibility to fungal colonization [20,21]. Non-immunological factors should also be considered. Reduced manual dexterity resulting from chronic joint involvement may compromise oral hygiene practices, whereas xerostomia, prolonged antibiotic exposure, and alterations in the oral and intestinal microbiota may further promote *Candida* colonization independently of autoimmune inflammation [23,24,25]. Because information on medication history, oral *Candida* colonization, intestinal permeability, and oral health status was unavailable in this study, the relative contribution of these factors could not be assessed and should be investigated in future studies.

Although serum IL-17A concentrations were significantly higher in *C. albicans*-positive RA patients than in *C. albicans*-negative patients, these findings should be interpreted with caution. Elevated IL-17A concentrations may reflect the underlying inflammatory activity of RA itself rather than a direct consequence of fungal DNA positivity. Disease activity indices, including the Disease Activity Score in 28 joints (DAS28), inflammatory biomarkers such as C-reactive protein (CRP) and erythrocyte sedimentation rate (ESR), and serological markers including rheumatoid factor (RF) and anti-citrullinated protein antibodies (ACPA), were not available for all participants and therefore could not be included in the present analyses. Consequently, potential confounding by disease severity cannot be excluded, and the observed association should not be interpreted as evidence that fungal DNA detection independently increases IL-17A production [2,13,26].

Another important limitation of this study is the use of conventional qualitative PCR rather than quantitative real-time PCR (qPCR). Although conventional PCR is a well-established and reliable method for detecting fungal DNA, it does not provide information regarding fungal burden. Consequently, it was not possible to determine whether the higher serum IL-17A concentrations observed in this study were associated with an increased fungal DNA load or simply with the presence of detectable fungal DNA. Quantitative real-time PCR, digital PCR, and next-generation sequencing technologies could provide more comprehensive information on fungal burden, fungal viability, and host–fungus interactions and should therefore be considered in future investigations [27,28,29].

Several additional limitations warrant consideration. First, the cross-sectional design precludes assessment of the temporal relationship between fungal DNA detection and inflammatory activity. Longitudinal studies are needed to determine whether fungal DNA positivity precedes changes in IL-17A concentrations or develops as a consequence of immune dysregulation associated with RA. Second, information on disease activity indices (DAS28), inflammatory markers (CRP and ESR), serological biomarkers (RF and ACPA), medication history, and oral or intestinal *Candida* colonization was unavailable for the study cohort. These factors may influence both fungal colonization and cytokine production and therefore represent potential confounding variables. Third, fungal DNA was assessed only in peripheral blood. Simultaneous analysis of oral, gastrointestinal, or fecal specimens together with blood samples would provide a more comprehensive understanding of the relationship between mucosal colonization, microbial translocation, and systemic immune responses [18,20,30].

Despite these limitations, this study has several notable strengths. To our knowledge, few clinical studies have simultaneously evaluated peripheral blood *Candida albicans* DNA and circulating IL-17A concentrations in patients with rheumatoid arthritis. By combining molecular detection using conventional PCR with quantitative cytokine measurement by ELISA, this study provides complementary molecular and immunological data from the same patient cohort. Furthermore, the inclusion of an age- and sex-matched healthy control group enabled direct comparisons of fungal DNA positivity and serum IL-17A concentrations between individuals with and without autoimmune disease.

In summary, the findings of this study indicate that *Candida albicans* DNA was detected more frequently in patients with rheumatoid arthritis than in healthy controls and that fungal DNA positivity was associated with higher circulating IL-17A concentrations. However, these findings should be interpreted as statistical associations rather than evidence of a causal relationship. Future multicenter prospective studies incorporating quantitative fungal detection methods, comprehensive assessments of disease activity, detailed medication histories, analyses of mucosal *Candida* colonization, and multi-cytokine profiling will be required to clarify the biological significance of fungal DNAemia and its relationship with immune dysregulation in rheumatoid arthritis.

## 4. Materials and Methods

**Study Design and Participants.** This case–control study was conducted to investigate the association between the presence of *Candida albicans* DNA in peripheral blood and serum interleukin-17A (IL-17A) concentrations in patients with rheumatoid arthritis (RA). A total of 100 participants were enrolled, including 50 patients diagnosed with RA and 50 apparently healthy volunteers who served as the control group. The study was conducted between June 2025 and February 2026 at Al-Husayniya Hospital, Baghdad, Iraq, in collaboration with the Department of Biology, College of Science, Al-Nahrain University, Baghdad, Iraq [23].

Patients with RA were diagnosed by experienced rheumatologists according to the 2010 American College of Rheumatology/European Alliance of Associations for Rheumatology (ACR/EULAR) Classification Criteria for Rheumatoid Arthritis [24]. Healthy controls were frequency-matched to the RA group by age and sex and were recruited from individuals undergoing routine health examinations. None of the control participants had a history of autoimmune disease, chronic inflammatory disorders, malignancy, immunodeficiency, systemic fungal infection, or any other condition known to affect immune function [25].

The primary objective of this study was to determine the prevalence of peripheral blood *Candida albicans* DNA in patients with RA and healthy controls using conventional polymerase chain reaction (PCR), quantify serum IL-17A concentrations using enzyme-linked immunosorbent assay (ELISA), and evaluate the association between fungal DNA positivity and circulating IL-17A levels [26,27]. Given the observational case–control design, the study was intended to identify statistical associations rather than establish causal relationships between fungal DNA detection and inflammatory responses [23].

Participants were eligible for inclusion if they met the following criteria:

- Age ≥ 18 years.
- Diagnosis of rheumatoid arthritis according to the 2010 ACR/EULAR classification criteria (RA group).
- Apparently healthy individuals without autoimmune diseases (control group).
- Ability and willingness to provide written informed consent.

Participants were excluded if any of the following conditions were present:

- Pregnancy or breastfeeding.
- Active bacterial, viral, or systemic fungal infection.
- History of malignancy.
- Chronic liver or chronic kidney disease.
- Other autoimmune or immunodeficiency disorders.
- Refusal or inability to provide informed consent.

All laboratory analyses, including molecular detection of *Candida albicans* DNA and measurement of serum IL-17A concentrations, were performed under standardized laboratory conditions in accordance with the manufacturers’ instructions.

**Ethical Approval.** The study protocol was reviewed and approved by the Institutional Review Board (IRB) of Nasiriyah Heart Hospital, Dhi Qar Health Directorate, Nasiriyah, Iraq. The study was conducted in accordance with the ethical principles of the Declaration of Helsinki and its subsequent amendments.

Before enrollment, all participants were informed about the objectives of the study, the procedures involved, the potential risks and benefits, data confidentiality, and their right to withdraw from the study at any time without affecting their medical care. Written informed consent was obtained from all participants before blood sample collection and laboratory analyses. Personal information was anonymized prior to statistical analysis to ensure participant confidentiality.

**Blood Sample Collection and Sample Processing.** Peripheral venous blood samples were collected from all participants under aseptic conditions by trained healthcare personnel. Approximately 5–10 mL of venous blood was obtained from each participant using sterile disposable needles and vacuum blood collection tubes. Immediately after collection, each blood sample was divided into two aliquots. One aliquot was transferred into EDTA-containing tubes for genomic DNA extraction and molecular detection of *Candida albicans*, whereas the second aliquot was collected into serum separator tubes for the measurement of serum IL-17A concentrations.

Serum samples were allowed to clot at room temperature for approximately 30 min and were subsequently centrifuged at 3000× *g* for 10 min. The separated serum was carefully transferred into sterile nuclease-free microcentrifuge tubes and stored at −80 °C until analysis. Samples showing visible hemolysis or insufficient serum volume were excluded from cytokine analysis.

Whole blood samples collected in EDTA tubes were processed for DNA extraction as soon as possible after collection. When immediate processing was not feasible, samples were stored at 4 °C for no longer than 24 h before DNA extraction to minimize nucleic acid degradation.

To ensure sample integrity, all specimens were assigned unique identification codes immediately after collection. Laboratory personnel performing the PCR and ELISA analyses were blinded to the clinical status of the participants throughout the analytical procedures. Blood collection, serum preparation, and specimen handling were carried out in accordance with internationally accepted laboratory guidelines for clinical immunological and molecular investigations [28,29,30,31].

**Genomic DNA Extraction.** Genomic DNA was extracted from peripheral whole blood samples using the DNeasy Blood & Tissue Kit (Qiagen, Hilden, Germany) according to the manufacturer’s instructions. Briefly, whole blood samples were lysed with Proteinase K and the appropriate lysis buffer, after which the released DNA was bound to silica membrane spin columns. The columns were subsequently washed with the manufacturer’s washing buffers to remove proteins, salts, and other potential PCR inhibitors. Purified DNA was eluted in nuclease-free elution buffer and stored at −20 °C until further molecular analysis [32,33].

DNA concentration and purity were determined using a NanoDrop spectrophotometer (Thermo Fisher Scientific, Waltham, MA, USA) by measuring absorbance at 260 and 280 nm. DNA samples with an A260/A280 ratio between 1.8 and 2.0 were considered suitable for downstream PCR amplification. Samples with insufficient DNA concentration or suboptimal purity were either re-extracted or excluded from subsequent molecular analyses.

**Conventional PCR Detection of *Candida albicans*.** The presence of *Candida albicans* DNA in peripheral blood samples was determined by conventional polymerase chain reaction (PCR) using species-specific primers targeting the INT1 gene, which encodes an integrin-like cell surface protein and has been widely used for the molecular identification of *C. albicans* [34]. The primer sequences used in this study were as follows:
**Primer****Sequence (5′→3′)**INT1-FAATGGCAATCCCAACGTTCINT1-RTCCCAATAACAAACACAGGA

The expected amplicon size was 344 bp [34].

PCR amplification was performed in a final reaction volume of 25 µL containing 12.5 µL of 2× NucleoGene PCR Master Mix (NucleoGene Biotechnology, Ankara, Türkiye), 1.0 µL each of forward and reverse primers (10 pmol/µL), 2.0 µL of template DNA, and 8.5 µL of nuclease-free water.

PCR amplification was carried out using a Kyratec Palm-Cycler™ Thermal Cycler (Kyratec Pty Ltd., Mansfield, Queensland, Australia) under the following cycling conditions: an initial denaturation at 95 °C for 5 min, followed by 35 cycles of denaturation at 95 °C for 30 s, annealing at 55 °C for 30 s, and extension at 72 °C for 45 s, with a final extension at 72 °C for 5 min.

PCR products were separated by electrophoresis on 1.5% agarose gels prepared in 1× TAE buffer and stained with ethidium bromide (0.5 µg/mL). Electrophoresis was performed at 100 V for approximately 45 min. Amplified DNA fragments were visualized under ultraviolet illumination using a Gel Doc XR+ gel documentation system (Bio-Rad Laboratories, Hercules, CA, USA). A 100 bp DNA ladder (Thermo Fisher Scientific, Vilnius, Lithuania) was included in each electrophoresis run to estimate amplicon size.

Each PCR run included a confirmed *Candida albicans* DNA sample as a positive control and nuclease-free water in place of template DNA as a no-template negative control to monitor amplification efficiency and potential contamination. To minimize the risk of PCR contamination, DNA extraction, PCR master mix preparation, and post-amplification analyses were performed in physically separated work areas whenever possible [12,13].

Samples exhibiting a distinct band at approximately 344 bp were considered positive for *C. albicans* DNA. The gel image presented in the Section 2 is a representative electrophoretic image and is not intended to reflect the overall positivity rate observed in the study population.

Serum IL-17A concentrations were measured using a commercially available Quantikine^®^ Human IL-17A Sandwich Enzyme-Linked Immunosorbent Assay (ELISA) kit (Catalog No. D1700; R&D Systems, Minneapolis, MN, USA) according to the manufacturer’s instructions [35,36].

Before analysis, frozen serum samples were thawed at room temperature and gently mixed. All serum samples, standards, and quality control materials were analyzed in duplicate to minimize intra-assay variability. Briefly, 100 µL of standards and serum samples were added to microplate wells pre-coated with a monoclonal antibody specific for human IL-17A and incubated for the duration recommended by the manufacturer. After washing to remove unbound material, a biotin-conjugated detection antibody was added, followed by horseradish peroxidase (HRP)-conjugated streptavidin. Following additional washing steps, 3,3′,5,5′-tetramethylbenzidine (TMB) substrate solution was added to each well and incubated in the dark to allow color development. The enzymatic reaction was terminated using the supplied stop solution, and absorbance was measured at 450 nm using an ELx808™ Absorbance Microplate Reader (BioTek Instruments, Winooski, VT, USA) [37].

A standard calibration curve was generated using recombinant human IL-17A standards supplied with the kit, and cytokine concentrations were calculated according to the manufacturer’s instructions. Results were expressed as pg/mL. Samples with absorbance values outside the analytical range were reanalyzed following appropriate dilution. Internal quality control procedures were implemented throughout the assay to ensure analytical accuracy and reproducibility [35,37].

**Statistical Analysis.** Statistical analyses were performed using IBM SPSS Statistics version 26.0 (IBM Corp., Armonk, NY, USA) [38]. Continuous variables are presented as mean ± standard deviation (SD), whereas categorical variables are expressed as frequencies and percentages.

The normality of continuous variables was assessed using the Shapiro–Wilk test [39]. Variables showing a normal distribution were analyzed using the independent-samples *t*-test (for comparisons between two groups) or one-way analysis of variance (ANOVA) followed by Tukey’s post hoc test for multiple-group comparisons. When the assumption of normality was not satisfied, the Mann–Whitney *U* test or Kruskal–Wallis test was applied as appropriate [40].

Differences in categorical variables, including the frequency of *Candida albicans* DNA positivity, were analyzed using the chi-square (χ^2^) test or Fisher’s exact test, as appropriate.

Because *Candida albicans* DNA positivity is a dichotomous variable (positive/negative), the association between fungal DNA detection and serum IL-17A concentrations was assessed using the point-biserial correlation coefficient (*r_pb_*) rather than Pearson’s correlation coefficient [41]. Correlation strength was interpreted according to conventional criteria. All statistical tests were two-tailed, and a *p* value of <0.05 was considered statistically significant [42].

A post hoc power analysis was performed using G*Power version 3.1 (Heinrich Heine University Düsseldorf, Düsseldorf, Germany) to evaluate whether the sample size provided sufficient statistical power to detect differences between the study groups. The calculated statistical power exceeded 80%, indicating that the sample size was adequate for the primary outcome analysis [43].

## 5. Conclusions

This study demonstrated that *Candida albicans* DNA was detected significantly more frequently in patients with rheumatoid arthritis than in healthy controls. In addition, serum IL-17A concentrations were significantly higher in patients with RA, with the highest levels observed among individuals who tested positive for *C. albicans* DNA. Furthermore, point-biserial correlation analysis revealed a significant association between fungal DNA positivity and circulating IL-17A concentrations.

These findings support an association between peripheral blood *C. albicans* DNA detection and enhanced IL-17A-mediated immune responses in rheumatoid arthritis. However, because of the cross-sectional design and the use of conventional qualitative PCR, this study cannot determine whether fungal DNA positivity contributes to inflammatory activity or instead represents a consequence of immune dysregulation associated with RA. Therefore, the observed relationship should be interpreted as an association rather than evidence of a causal relationship.

Overall, these findings expand current knowledge of the potential interaction between fungal DNAemia and immune activation in rheumatoid arthritis and highlight the importance of considering fungal-related factors in future immunological research. Further multicenter prospective studies incorporating quantitative fungal detection methods, comprehensive assessments of disease activity, detailed medication histories, analyses of mucosal *Candida* colonization, and broader cytokine profiling are needed to clarify the biological and clinical significance of this association.

## Figures and Tables

**Table 1:** Baseline demographic and clinical characteristics of the study population.

	RA Patients (n = 50)	Healthy Controls (n = 50)	*p* Value
Age (years), mean ± SD	48.3 ± 10.1	46.7 ± 9.8	0.421
Female, n (%)	38 (76)	36 (72)	0.643
Disease duration (years), mean ± SD	7.2 ± 3.6	Not applicable	—
DAS28 score	Not available	Not applicable	—
CRP (mg/L)	Not available	Not available	—
ESR (mm/h)	Not available	Not applicable	—
RF status	Not available	Not applicable	—
ACPA status	Not available	Not applicable	—
Medication history	Not available	Not applicable	—

Data are presented as mean ± SD or n (%). Age was compared using Student’s *t*-test, and sex was compared using the chi-square test. Disease duration was recorded only for patients with rheumatoid arthritis. DAS28, CRP, ESR, RF, ACPA, and medication history were unavailable and were therefore not included in the statistical analyses. **Abbreviations:** RA, rheumatoid arthritis; SD, standard deviation; DAS28, Disease Activity Score in 28 joints; CRP, C-reactive protein; ESR, erythrocyte sedimentation rate; RF, rheumatoid factor; ACPA, anti-citrullinated protein antibodies.

**Table 2:** Prevalence of *Candida albicans* DNA Positivity in Study Groups.

Group	*C. albicans* Positive, n (%)	*C. albicans* Negative, n (%)	*p* Value
RA patients (n = 50)	19 (38%)	31 (62%)	0.001
Healthy controls (n = 50)	5 (10%)	45 (90%)	

Statistical significance was assessed using the chi-square (χ^2^) test. **Abbreviations:** RA, rheumatoid arthritis.

**Table 3:** Serum IL-17A Concentrations in the Study Groups.

Group	n	Mean IL-17A (pg/mL) ± SD	*p* Value
RA Patients (*C. albicans*+)	19	46.2 ± 8.7	<0.001 vs. HC
RA Patients (*C. albicans*−)	31	32.5 ± 6.4	<0.001 vs. HC
Healthy Controls	50	15.1 ± 5.2	—
*C. albicans*(+) vs. *C. albicans*(−) RA	—	—	<0.001

Serum IL-17A concentrations are presented as mean ± SD. Statistical comparisons were performed as described in the Section 4. **Abbreviations:** RA, rheumatoid arthritis; HC, healthy controls; SD, standard deviation.

## Data Availability

The datasets generated and/or analyzed during the current study are available from the corresponding author upon reasonable request.
